# Supplementary material for: Complexome profiling of the Chlamydomonas psb28 mutant reveals TEF5 as an early PSII assembly factor
Source: Plant Cell. 2025 Mar 17;37(6):koaf055. doi: 10.1093/plcell/koaf055 (PMC12164590; doi:10.1093/plcell/koaf055)
Supplement: koaf055_Supplementary_Data [file koaf055_supplementary_data.zip › psb28_interactive.zip!/plots/intensity/qualityPlots/peptides/_Cre17.g744897.t1.1.html]

### Cre17.g744897.t1.1

Trivial name:   
SeqCoverage [median]: 21.40  
Mass: 45.66 kDa  
  
 

| Sequence | MissedCleavage | Mass [Da] | wtIntensity | muIntensity | Score |
| --- | --- | --- | --- | --- | --- |
| AADLAALLR | 0 | 912.54 | 1014.34 | 0.00 | 119.29 |
| ALVADKPSLLTK | 1 | 1254.75 | 1559.29 | 3216.97 | 69.01 |
| AVAEAEAEEAGAEAQAGR | 0 | 1728.79 | 1704.05 | 893.43 | 81.43 |
| EAPPGAVVTPAAGGSGGR | 0 | 1549.78 | 0.00 | 387.69 | 71.24 |
| LAEALEVPR | 0 | 996.56 | 731.63 | 1236.78 | 83.27 |
| VLLVPAAELR | 0 | 1079.67 | 2094.71 | 0.00 | 71.38 |
| VSPATAQSLLASER | 0 | 1428.76 | 0.00 | 1914.09 | 74.96 |
| VVLNAPR | 0 | 767.47 | 0.00 | 1141.04 | 72.00 |
|  |  |  | 7104.0 | 8790.0 |  |
